# AI-Driven Data Analysis of Quantifying Environmental Impact and Efficiency of Shape Memory Polymers

**DOI:** 10.3390/biomimetics9080490

**Published:** 2024-08-14

**Authors:** Mattew A. Olawumi, Bankole I. Oladapo, Temitope Olumide Olugbade, Francis T. Omigbodun, David B. Olawade

**Affiliations:** 1 Computing, Engineering and Media, De Montfort University, Leicester LE1 9BH, UK; olawumisola13@gmail.com; 2 School of Science and Engineering, University of Dundee, Dundee DD1 4HN, UK; tolugbade001@dundee.ac.uk; 3 Wolfson School of Mechanical, Electrical and Manufacturing Engineering, Loughborough University, Loughborough LE11 3TU, UK; f.omigbodun@lboro.ac.uk; 4 Department of Allied and Public Health, School of Health, Sport and Bioscience, University of East London, London E16 2RD, UK; olawadedavid@gmail.com; 5 Department of Research and Innovation, Medway NHS Foundation Trust, Gillingham ME7 5NY, UK

**Keywords:** shape memory, AI-driven data analysis, medical implants, 4D printing applications, environmental sustainability

## Abstract

This research investigates the environmental sustainability and biomedical applications of shape memory polymers (SMPs), focusing on their integration into 4D printing technologies. The objectives include comparing the carbon footprint, embodied energy, and water consumption of SMPs with traditional materials such as metals and conventional polymers and evaluating their potential in medical implants, drug delivery systems, and tissue engineering. The methodology involves a comprehensive literature review and AI-driven data analysis to provide robust, scalable insights into the environmental and functional performance of SMPs. Thermomechanical modeling, phase transformation kinetics, and heat transfer analyses are employed to understand the behavior of SMPs under various conditions. Significant findings reveal that SMPs exhibit considerably lower environmental impacts than traditional materials, reducing greenhouse gas emissions by approximately 40%, water consumption by 30%, and embodied energy by 25%. These polymers also demonstrate superior functionality and adaptability in biomedical applications due to their ability to change shape in response to external stimuli. The study concludes that SMPs are promising sustainable alternatives for biomedical applications, offering enhanced patient outcomes and reduced environmental footprints. Integrating SMPs into 4D printing technologies is poised to revolutionize healthcare manufacturing processes and product life cycles, promoting sustainable and efficient medical practices.

## 1. Introduction

The demand for innovative materials enhancing sustainability and functionality in various industries has surged recently. This is particularly evident in the biomedical sector, where the environmental impact of traditional manufacturing processes and materials remains a significant concern [1,2,3]. Among the forefront of advancements are four-dimensional (4D) printing technologies that promise revolutionary changes in manufacturing dynamics and aim to reduce ecological footprints [4,5,6].

4D printing, an evolution of three-dimensional (3D) printing, introduces the dimension of time to printed objects, allowing them to change shape or function in response to external stimuli. This technology utilizes various smart materials capable of responding to changes in their environment, such as temperature, light, moisture, or magnetic fields [7,8,9]. The principal categories of these materials include shape memory polymers (SMPs), thermo-reactive polymer hydrogels, and other stimuli-responsive materials, each possessing unique characteristics that make them suitable for specific applications [10,11,12].

SMPs are pivotal in 4D printing because they can remember and switch between temporary and permanent shapes under stimuli [13,14,15]. This property is immensely beneficial for biomedical applications, such as minimally invasive surgical implants that can be inserted in a compact form and then expanded in the body, reducing patient trauma and recovery time [15,16,17].

Thermo-reactive polymer hydrogels, on the other hand, exhibit significant potential due to their sensitivity to temperature changes. These materials can undergo drastic but reversible changes in their network structure and volume in response to temperature shifts, making them ideal for drug delivery systems where controlled release is crucial [18,19,20]. Below their transition temperature, these hydrogels remain swollen and release encapsulated drugs or chemicals as they contract when heated [21,22,23]. In addition, other 4D printing materials include biomimetic composites, magneto-active substances, and light-responsive materials, each opening new possibilities for application-specific innovations.

**Figure 1:**
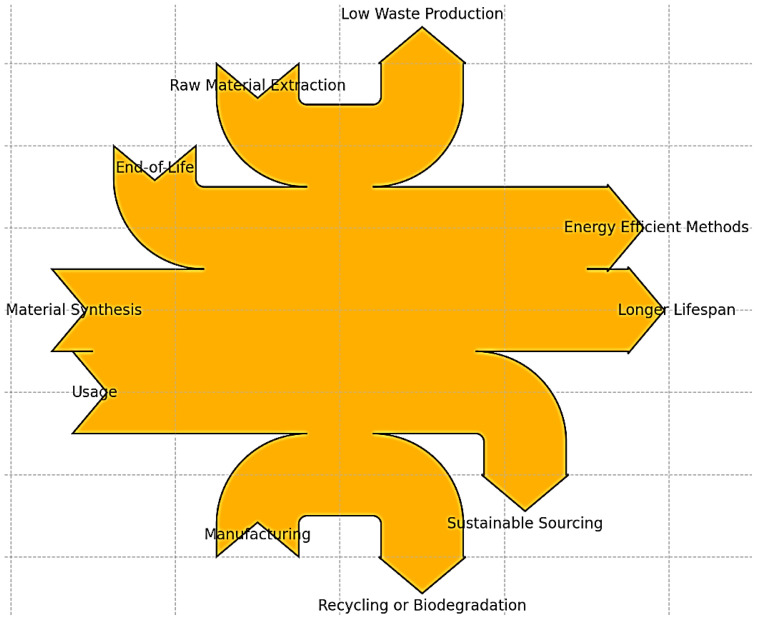
Life Cycle Analysis of SMPs Description: a flowchart illustrating the life cycle stages of SMPs from production to disposal.

The primary objective of this review is to comprehensively assess the sustainability, functionality, and potential environmental impacts of SMPs within the context of 4D printing technologies, mainly focusing on biomedical applications. The review aims to analyze the comparative ecological benefits of SMPs against traditional metallic and polymer materials used in medical implants, exploring metrics such as carbon footprint, embodied energy, and water usage. By investigating these aspects, the review seeks to elucidate the advantages and challenges of adopting SMPs in the biomedical sector, highlighting their ability to enhance medical practices through improved patient outcomes and reduced environmental impact. This study also aims to provide insights into the future development and integration of SMPs in 4D printing, paving the way for more sustainable, efficient, and patient-centered medical applications. The flowchart in Figure 1 visually represents the key stages and environmental considerations involved in the life cycle of SMPs.

**Figure 2:**
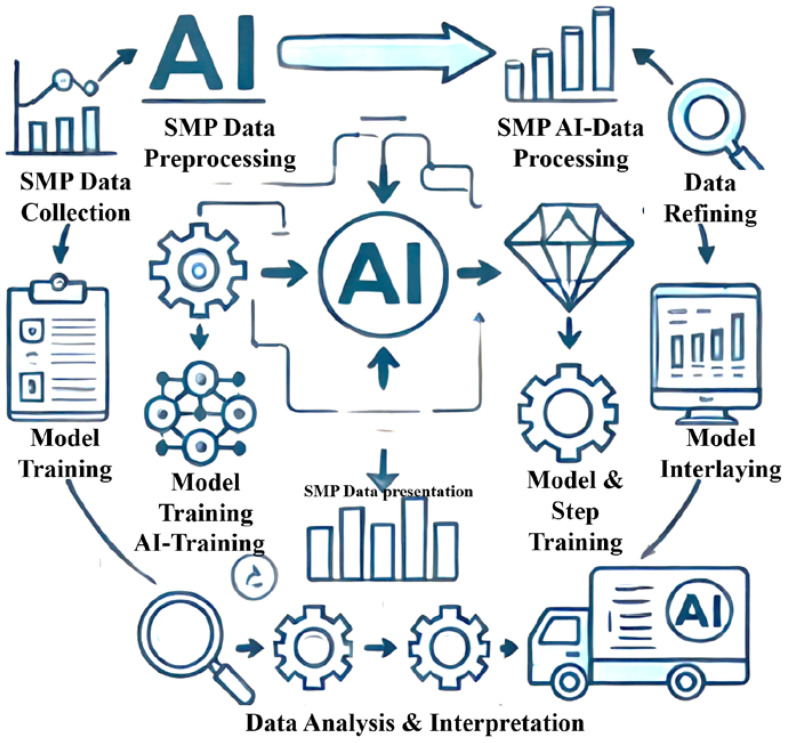
AI-Driven Data Analysis Workflow Description: a flowchart illustrating the workflow of AI-driven data analysis used in the study. Content: steps including data collection, preprocessing, model training, analysis, and interpretation.

The primary objective of this research is to explore the sustainability and functional potential of SMPs in biomedical applications, specifically focusing on their integration into 4D printing technologies. The study aims to assess the environmental impacts of SMPs by comparing their carbon footprint, embodied energy, and water consumption against traditional metallic and polymer materials used in medical implants. Additionally, the research seeks to elucidate the advantages of SMPs in terms of their unique properties, such as the ability to change and recover shape in response to external stimuli, which can significantly enhance the functionality and adaptability of biomedical implants. Through this comprehensive analysis, the research intends to demonstrate how SMPs can contribute to more sustainable medical practices and improved patient outcomes, marking a significant step forward in biomedical engineering. The increasing demand for sustainable materials in the biomedical industry has spurred research into alternative materials and manufacturing techniques. Shape memory polymers offer unique properties and environmental advantages, making them promising candidates for biomedical applications. By exploring the sustainability potential of SMPs and investigating the applications of 4D printing, this research aims to contribute to developing more sustainable and environmentally conscious biomedical practices. Figure 2 shows the workflow of AI-driven data analysis. The flowchart includes steps such as data collection, preprocessing, model training, analysis, and interpretation, with arrows showing the flow between steps and brief descriptions for each step. The design is clean and professional, suitable for inclusion in a research manuscript.

## 2. Sustainability Potential of Shape Memory Polymers

**Figure 3:**
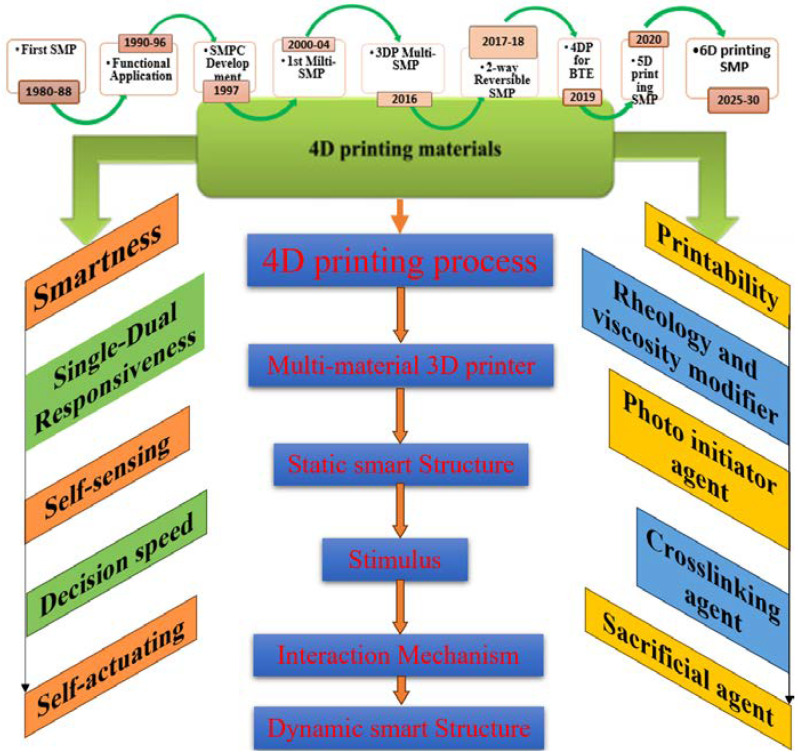
The progress of additive manufacturing technologies led to 4D printing and material processing.

SMPs have gained significant attention recently due to their unique properties and potential for sustainable applications in various industries, including biomedical engineering. These polymers can change their shape in response to external stimuli, such as temperature, light, or moisture, and then recover their original shape when the stimulus is removed. This shape memory effect, coupled with other advantageous properties, makes SMPs a promising choice for sustainable materials in biomedical applications [22,23,24]. One of the critical aspects of SMPs that contributes to their sustainability potential is their lower environmental impact compared to traditional metallic and polymer materials used in biomedical implants. The production processes of conventional materials, such as cobalt-chromium and titanium alloys, typically involve energy-intensive procedures, including mining, extraction, refinement, and manufacturing [24,25,26]. These processes contribute to greenhouse gas emissions, energy consumption, and water usage, thereby increasing the environmental footprint. SMPs can be synthesized using more energy-efficient and environmentally friendly methods (Figure 3). Some SMPs can be derived from renewable resources or biodegradable polymers, reducing reliance on fossil fuels and lowering carbon emissions [26,27,28]. SMP production can contribute to a more circular economy by utilizing sustainable feedstocks, where materials are derived from renewable sources and can be recycled or biodegraded at the end of their lifespan [28,29,30].

Additionally, the unique properties of SMPs, such as their deformability and biocompatibility, offer further sustainability benefits. SMPs can be designed to have mechanical properties that closely mimic natural tissues, providing an optimal environment for cell growth and tissue regeneration [31,32,33]. This characteristic reduces the need for multiple surgeries and enhances healing, improving patient outcomes and reducing healthcare costs. Using SMPs in tissue engineering and regenerative medicine can potentially contribute to a more sustainable healthcare system by minimizing the need for additional interventions and resources [34,35,36].

Furthermore, SMPs have the potential to enable minimally invasive surgeries and reduce the environmental impact associated with implantation procedures. Due to their shape memory effect, SMP-based medical devices can be implanted in a folded or contracted state, requiring smaller incisions and reducing the procedure’s invasiveness [36,37,38,39]. This results in shorter recovery times, reduced patient discomfort, and decreased healthcare resource utilization. Minimally invasive surgeries benefit the patients and contribute to sustainability efforts by minimizing resource consumption and reducing the surgical process’s environmental burden [39,40,41]. The advent of 3D printing and 4D printing technologies has further enhanced the sustainability potential of SMPs in biomedical applications. These additive manufacturing techniques allow for the precise fabrication of complex structures, including customized implants, using fewer raw materials and generating less waste than traditional manufacturing methods [42,43,44]. Creating patient-specific designs with optimized geometries improves implant performance and reduces material waste and energy consumption. 4D printing, which adds the dimension of time to 3D-printed structures, enables the creation of adaptive and responsive implants that can actively interact with the surrounding environment [45,46,47]. By incorporating SMPs into 4D printing processes, medical devices can be biomimetics designed to dynamically change their shape, properties, or functionality in response to physiological conditions. This capability can potentially enhance the longevity and performance of implants, reducing the need for frequent replacements and minimizing material waste.

**Figure 4:**
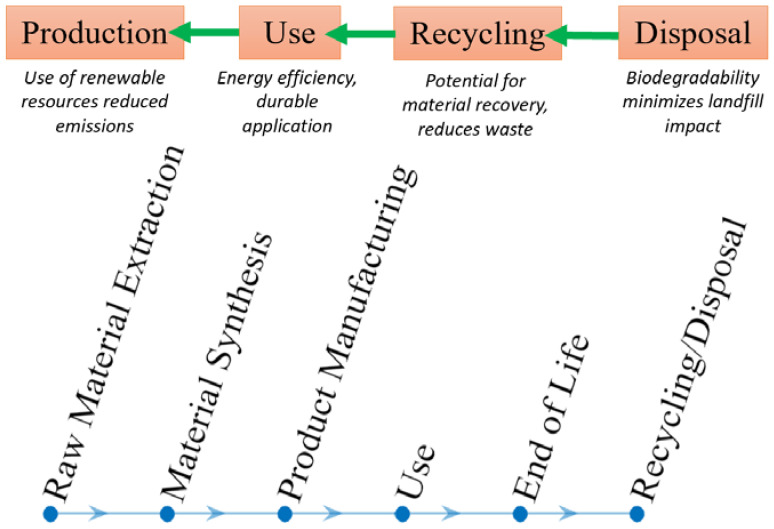
SMP life cycle analysis is a flowchart illustrating the stages from production to disposal of SMPs, highlighting key environmental considerations at each step.

In conclusion, shape memory polymers offer significant sustainability potential in biomedical applications. Their lower environmental impact, deformability, biocompatibility, and advancements in additive manufacturing techniques position SMPs as promising sustainable materials for biomedical implants. SMPs can improve patient outcomes, reduce healthcare costs, and minimize environmental footprints. Further research and development in SMPs, along with the continued advancements in 3D printing and 4D printing technologies, are crucial to fully realize the sustainability potential of these innovative materials in the biomedical field. Figure 4 shows a flowchart illustrating the life cycle analysis of SMPs. It shows the stages from production to disposal, with each step highlighting critical environmental considerations. This visualization aids in understanding the ecological impacts and management practices associated with SMPs through their life cycle.

## 3. Biological Applications and Management Processes

SMPs have demonstrated significant potential for various biological applications due to their unique properties, shape memory effect, responsiveness to stimuli, and biocompatibility [48,49,50]. This section explores the various biological applications of SMPs and discusses the management processes associated with their utilization in biomedical settings.

### 3.1. Tissue Engineering

Tissue engineering is a rapidly evolving field that aims to regenerate or replace damaged or diseased tissues using a combination of cells, biomaterials, and growth factors. SMPs have emerged as promising materials for tissue engineering applications due to their ability to mimic the mechanical properties of natural tissues and their compatibility with biological systems. One key advantage of SMPs in tissue engineering is their shape memory effect, which allows them to change their shape in response to external stimuli, such as temperature or moisture [51,52,53]. This property enables the fabrication of scaffolds that can be compressed for minimally invasive implantation and then recover their original shape within the body. The shape recovery process provides mechanical support to the surrounding tissues and promotes cell adhesion, migration, and proliferation.

**Figure 5:**
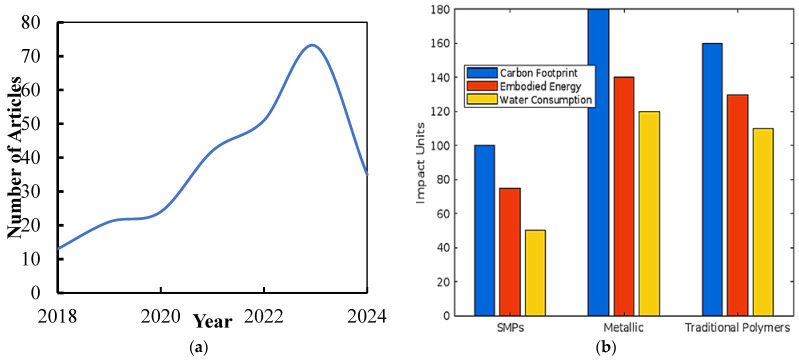
(**a**) Detailed insights and comparison of multiple related papers for research query on “4D printing shape memory polymers”; (**b**) Comparative Environmental Impact: a diagram showing the carbon footprint, embodied energy, and water consumption of SMPs compared to traditional metallic and polymer materials used in medical implants.

SMP-based scaffolds can be tailored to match the mechanical properties of different tissues, promoting proper cell alignment, differentiation, and tissue regeneration [54,55,56]. The porous structure of SMP scaffolds allows for the diffusion of nutrients and waste products, facilitating cell viability and tissue integration. Moreover, the surface properties of SMPs can be modified to promote cell adhesion and guide tissue growth, enhancing the overall performance of the engineered tissue constructs [57,58,59]. Using SMPs in tissue engineering also offers the potential for dynamic tissue regeneration. By incorporating stimuli-responsive elements into the SMPs, such as drug-releasing capabilities or growth factor gradients, it is possible to create scaffolds that can provide localized and controlled stimuli to promote tissue regeneration. This dynamic aspect of SMP-based scaffolds has the potential to enhance the healing process and facilitate the regeneration of complex tissues. Figure 5a shows detailed insights and comparison of multiple related papers for research query on “4D printing shape memory polymers” on the National Institutes of Health (NIH) Search. Figure 5b displays each material type’s carbon footprint, embodied energy, and water consumption. The comparative environmental impact diagram shows the carbon footprint, embodied energy, and water consumption of SMPs compared to traditional metallic and polymer materials used in medical implants.

### 3.2. Drug Delivery Systems

SMPs have also shown promise in the development of novel drug delivery systems. The shape memory effect of SMPs allows for the controlled release of drugs or therapeutic agents in response to specific stimuli, such as temperature, pH, or light. This property enables the design of smart drug delivery systems that can release drugs at the desired site and time, improving treatment efficacy and minimizing side effects [60,61,62,63,64,65,66,67,68,69,70,71,72]. Depending on the specific application requirements, SMP-based drug delivery systems can be fabricated in various forms, including films, microspheres, or hydrogels. The stimuli-responsive nature of SMPs allows for the on-demand release of drugs, ensuring optimal therapeutic concentrations are achieved at the target site. This controlled release mechanism can improve patient compliance and reduce the frequency of drug administration. Furthermore, the biocompatibility and tunable properties of SMPs make them suitable for encapsulating and protecting sensitive drugs or biologics [73,74]. The ability of SMPs to undergo reversible shape changes also enables the development of injectable drug delivery systems, where the material can be delivered in a compact form and then expand to release the drug at the desired location.

### 3.3. Biomedical Implants

**Figure 6:**
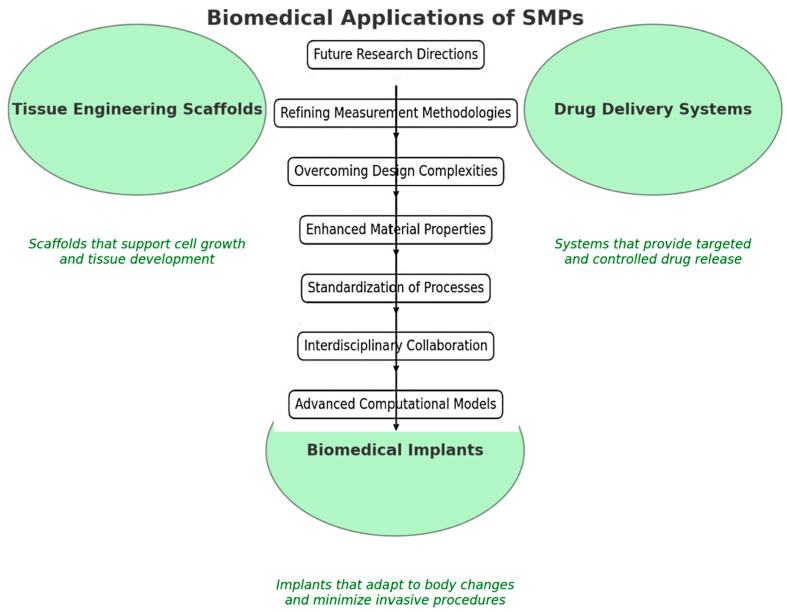
Biomedical applications of SMPs used in various biomedical applications, such as tissue engineering scaffolds, drug delivery systems, and biomedical implants.

SMPs have gained attention as potential materials for biomedical implants due to their unique properties and biocompatibility. The shape memory effect of SMPs allows for the development of implants that can be delivered in a compact form and then recover their intended shape once implanted. This feature enables minimally invasive surgical procedures, reducing patient trauma and recovery time. SMP-based implants can be biomimetically designed to have mechanical properties that closely match those of natural tissues, reducing stress shielding and improving long-term performance [74,75,76]. The biocompatibility of SMPs ensures minimal adverse reactions or inflammatory responses when in contact with biological systems. Additionally, SMPs can be surface-modified to enhance cell adhesion, integration, and tissue regeneration around the implant site. One notable application of SMP-based implants is in the field of cardiovascular devices. SMP stents, for example, can be delivered in a compressed form and deployed at the desired location within blood vessels, providing mechanical support and preventing vessel occlusion. The ability of SMP stents to self-expand to their predetermined shape ensures proper vessel wall apposition and reduces the risk of restenosis. Figure 6 shows the biomedical applications of SMPs. This visualization includes tissue engineering scaffolds, drug delivery systems, and biomedical plants, with descriptions for each application highlighted.

### 3.4. Management Processes

The successful utilization of SMPs in biological applications requires careful consideration of various management processes, including material selection, fabrication techniques, sterilization methods, and quality control measures. Material selection plays a crucial role in determining the performance and biocompatibility of SMPs in biomedical applications. Factors such as mechanical properties, degradation rate, surface characteristics, and biocompatibility must be carefully evaluated when selecting SMPs for specific applications [77,78,79]. The potential risks of releasing degradation by-products or leachable substances should also be assessed to ensure patient safety. Fabrication techniques for SMP-based biomedical devices must be optimized to achieve the desired shape memory properties, mechanical strength, and dimensional accuracy. Techniques such as 3D printing, electrospinning, and solvent casting are commonly employed to fabricate SMP scaffolds, films, or microstructures with precise control over geometry and porosity [80,81,82].

**Figure 7:**
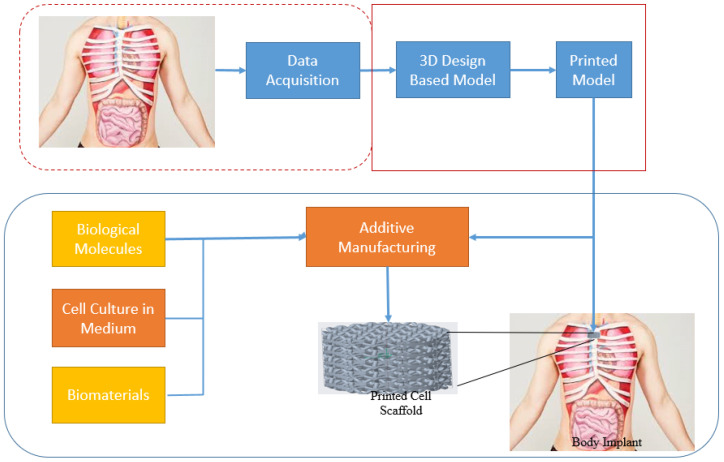
Fabric engineering textiles and bone implants may be made using these techniques [20,21,22].

Sterilisation of SMP-based devices is essential to eliminate microorganisms and minimize the risk of infections. Sterilization methods, such as gamma irradiation, ethylene oxide gas sterilization, or autoclaving, should be validated to ensure their compatibility with the specific SMP materials and fabrication techniques. Quality control measures should be implemented throughout the manufacturing process to ensure the consistency and reliability of SMP-based biomedical devices. This includes rigorous testing of mechanical properties, shape memory behavior, biocompatibility, and degradation characteristics [83,84,85]. Compliance with relevant regulatory standards and guidelines is also critical to ensure the safety and efficacy of SMP-based products. SMPs are promising for many biological applications, including tissue engineering, drug delivery systems, and biomedical implants. The shape memory effect, responsiveness to stimuli, and biocompatibility of SMPs enable the development of innovative and dynamic biomaterials. However, successfully utilizing SMPs in biomedical settings requires careful consideration of material selection, fabrication techniques, sterilization methods, and quality control measures (Figure 7). With further research and development, SMPs have the potential to revolutionize the field of biomedicine and contribute to improved patient care and outcomes.

## 4. Environmental Impact Assessment

As the world becomes increasingly aware of the environmental consequences of human activities, assessing environmental impacts has gained significant importance. This section focuses on the environmental impact assessment of SMPs, considering their production, use, and disposal stages. Understanding the potential ecological implications of SMPs is crucial for developing sustainable strategies and mitigating any adverse effects.

### 4.1. Production Stage

The production of SMPs involves several processes, including synthesizing polymer precursors, polymerization, and post-processing steps. These stages may include using various chemicals, energy-intensive operations, and generating waste streams. It is essential to assess the environmental impacts associated with these processes to identify potential areas for improvement. One aspect to consider is the raw materials used in SMP production. Traditional polymer precursors are typically derived from fossil fuels, contributing to greenhouse gas emissions and depletion of natural resources. However, efforts are being made to explore alternative feedstocks, such as bio-based materials or renewable resources, to reduce the environmental footprint of SMP production [83,84,85].

The energy consumption during SMP synthesis and processing also needs to be evaluated. Energy-intensive processes, such as heating, cooling, and solvent evaporation, can contribute to greenhouse gas emissions and air pollution [85,86,87]. Implementing energy-efficient practices, utilizing renewable energy sources, and optimizing process parameters can help minimize the environmental impact of SMP production. Waste generation and management are other crucial aspects to consider [87,88]. Disposing of chemical waste and by-products from SMP manufacturing processes can pose environmental risks if not properly managed. Implementing waste reduction strategies, such as recycling or reusing materials, and appropriate treatment and disposal methods for any generated waste streams is vital [89,90].

### 4.2. Life Cycle Assessment

A life cycle assessment (LCA) evaluates a product or system’s environmental aspects and potential impacts throughout its life cycle, from raw material extraction to final disposal. LCA considers multiple ecological indicators, such as greenhouse gas emissions, energy consumption, water use, and waste generation. By quantifying and analyzing these impacts, LCA provides a holistic view of the environmental performance of SMPs and helps identify areas for improvement [90,91,92].

**Figure 8:**
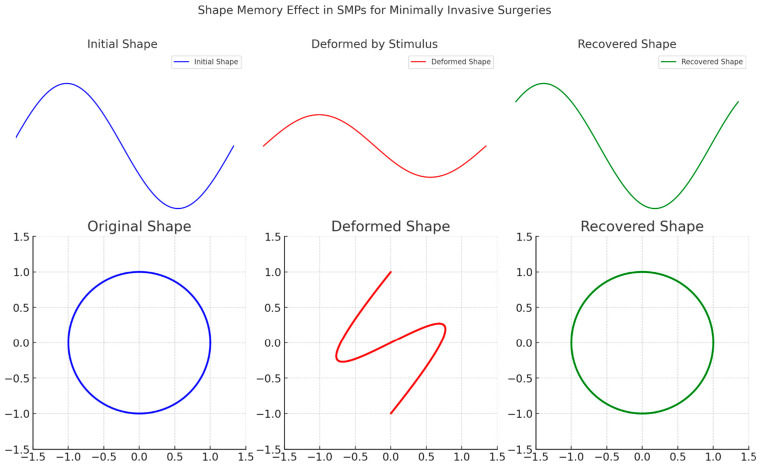
Shape memory effect in SMPs: a series of images or diagrams demonstrating how SMPs can change and recover their shape in response to external stimuli, illustrating their applications in minimally invasive surgeries.

Furthermore, LCA enables comparisons of different material options and technologies, allowing decision-makers to make informed choices based on environmental considerations. It helps identify trade-offs and potential hotspots in the life cycle of SMPs, guiding the development of sustainable practices and policies. In conclusion, the environmental impact assessment of SMPs is crucial for understanding and mitigating their potential ecological implications. Assessing the production, use, and end-of-life stages of SMPs allows for identifying areas for improvement and developing sustainable strategies. Employing LCA provides a comprehensive view of the environmental performance of SMPs and facilitates informed decision-making. Addressing the environmental challenges associated with SMPs makes it possible to harness their full potential while minimizing adverse environmental effects. Figure 8 demonstrates the unique properties of SMPs that make them suitable for applications like minimally invasive surgeries, where they can be inserted in a compact form and then expanded to perform their function inside the body.

## 5. Thermo-Reactive Polymer Hydrogels and Applications

Thermo-reactive polymer hydrogels are a class of materials that exhibit unique properties in response to changes in temperature. These hydrogels undergo reversible phase transitions, transforming their physical state or properties based on temperature variations. This section explores the characteristics of thermo-reactive polymer hydrogels and their diverse applications in various fields. Thermo-reactive polymer hydrogels typically comprise a polymer network dispersed in a water-rich environment. The polymer chains within the hydrogel structure can undergo reversible coil-to-globule transitions or exhibit changes in swelling behavior in response to temperature changes. One commonly used thermo-reactive hydrogel polymer is poly N-isopropyl acrylamide (pNIPAM) [92,93,94]. Below its lower critical solution temperature (LCST) of approximately 32 °C, pNIPAM hydrogels exhibit a swollen state due to increased hydrophilicity. However, above the LCST, the hydrogels transition to a collapsed or globular state, leading to decreased swelling and changes in mechanical properties [94,95,96]. The reversibility of the thermo-responsive behavior makes these hydrogels attractive for numerous applications. The hydrogel can repeatedly transition between swollen and collapsed states with temperature changes, allowing for control over its properties and functionality.

### 5.1. Applications of Thermo-Reactive Polymer Hydrogels

#### 5.1.1. Drug Delivery Systems

Thermo-reactive polymer hydrogels have been extensively explored as drug delivery systems. By incorporating drugs or therapeutic agents into the hydrogel matrix, controlled release can be achieved by exploiting the temperature-dependent swelling behavior. Below the LCST, the hydrogel swells, allowing for the encapsulation and entrapment of drugs within its structure. When exposed to temperatures above the LCST, the hydrogel collapses, expelling or releasing the encapsulated drugs. This temperature-triggered release mechanism offers precise control over drug delivery, enabling targeted and localized therapies. Furthermore, the ability of thermo-reactive hydrogels to respond to external stimuli, such as temperature, provides opportunities for on-demand drug release [96,97,98]. By applying heat externally or locally, the hydrogel can be triggered to release drugs at specific sites or time intervals, enhancing therapeutic efficacy and minimizing side effects.

#### 5.1.2. Tissue Engineering and Regenerative Medicine

**Figure 9:**
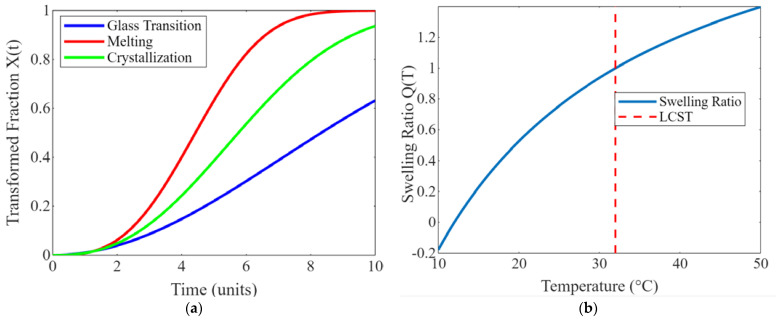
(**a**) Phase transformation kinetics of SMPs; (**b**) thermo-responsive behavior of hydrogels.

Thermo-reactive polymer hydrogels have also found applications in tissue engineering and regenerative medicine. The ability of these hydrogels to undergo reversible phase transitions in response to temperature changes enables the encapsulation and delivery of cells or bioactive molecules for tissue regeneration [98,99,100]. The hydrogel’s transition from a swollen state to a more compact state facilitates cell encapsulation within the matrix, promoting cell adhesion, proliferation, and differentiation. Additionally, the hydrogel’s porous structure allows for the diffusion of nutrients and waste products, creating an environment conducive to tissue growth and regeneration. Moreover, thermo-reactive polymer hydrogels can be engineered to provide mechanical support and mimic the native extracellular matrix (ECM). By tailoring the hydrogel composition and crosslinking density, it is possible to create scaffolds with the desired mechanical properties and porosity, facilitating cell migration and tissue integration [100,101,102]. Figure 9 shows the heat transfer in SMPs during the shape recovery. The heat maps display the temperature distribution at different time points, illustrating the temperature gradients and heat flow within the SMP over time. This visualization helps in understanding the thermal behavior of SMPs during their recovery process.

### 5.2. Future Perspectives Polymer Hydrogels

Thermo-reactive polymer hydrogels hold significant promise in various fields, including drug delivery, tissue engineering, and robotics. As researchers continue to explore their properties and applications, several areas of future development can be anticipated. Further advancements in hydrogel synthesis and engineering techniques will enable the tailoring of hydrogel properties, such as LCST, mechanical strength, and degradation rates. This will allow for the design of hydrogels with precise temperature responsiveness and enhanced functionality. Moreover, integrating additional stimuli-responsive elements, such as pH or light responsiveness, with thermo-reactive hydrogels will broaden their range of applications and enable more sophisticated control over material properties and behaviors [102,103,104].

**Figure 10:**
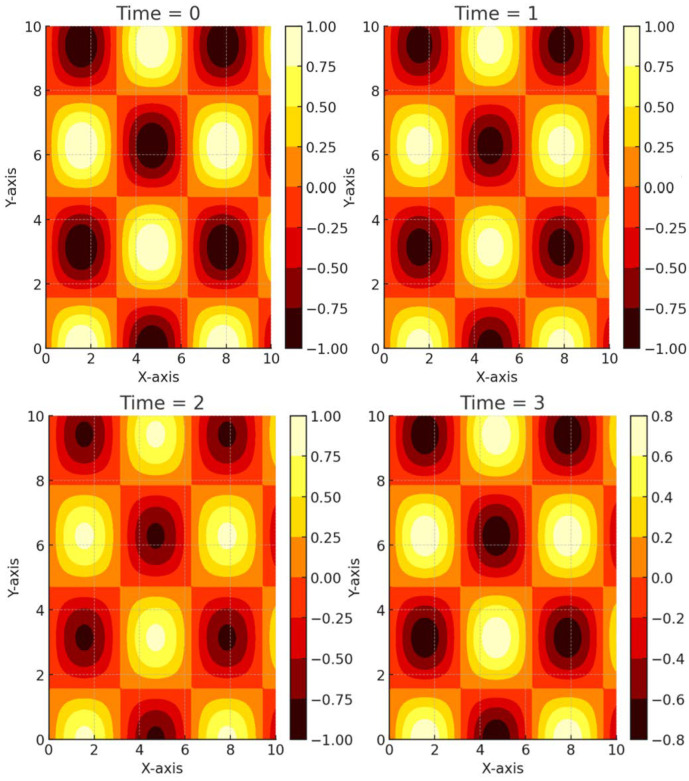
Heat transfer in SMPs with change in time.

Combining thermo-reactive hydrogels with bioactive factors and scaffold materials will contribute to developing complex tissue constructs with improved regenerative capabilities in tissue engineering. Additionally, using thermo-reactive hydrogels in soft robotics will lead to advancements in adaptive and autonomous systems, enabling the creation of robots with enhanced agility, flexibility, and responsiveness. In conclusion, thermo-reactive polymer hydrogels offer unique properties and capabilities that make them attractive for various applications. Their temperature-responsive behavior allows precise control over material properties, drug release, tissue regeneration, and actuation. As research advances in this field, thermo-reactive hydrogels hold great potential for addressing challenges in drug delivery, tissue engineering, and soft robotics, leading to innovative solutions and improved outcomes in various domains [104,105,106]. Figure 10 showcases an infographic of the applications of 4D printing. It includes sections for minimally invasive implants, drug delivery systems, tissue engineering, and soft robotics, each with icons and brief descriptions. The design uses vibrant colors and modern elements to make it visually appealing. A heat map or contour plot shows the temperature distribution in an SMP during the shape recovery process. A visualization of temperature gradients and heat flow within the SMP over time is included.

## 6. Biomedical Considerations for Biomimetics

Biomedical applications of SMPs have gained significant attention in recent years due to their unique properties and potential in various medical fields. This section explores the specific considerations and advancements in SMPs for biomedical applications, including tissue engineering, bioprinting, and implantable devices. Tissue engineering aims to create functional tissues or organs by combining cells, scaffolds, and bioactive factors. SMPs have emerged as promising materials for tissue engineering due to their ability to undergo reversible shape changes and compatibility with biological systems [106,107,108].

One key advantage of SMPs in tissue engineering is their shape memory effect, which allows them to adapt to complex anatomical shapes and maintain structural integrity. This property is precious in creating scaffolds or implants that can be easily inserted into the body in a compact form and then recover their original shape once implanted. SMP scaffolds can be biomimetics designed to provide mechanical support, guide cell growth, and promote tissue regeneration. By incorporating bioactive molecules or cells within the SMP matrix, controlled release of growth factors or signaling molecules can be achieved, enhancing cell proliferation, differentiation, and tissue integration. Furthermore, SMPs can mimic the mechanical properties of native tissues, offering a more biomimetic environment for cell growth and tissue development. By tuning the composition and crosslinking density of SMPs, their stiffness, elasticity, and degradation rates can be tailored to match specific tissue requirements.

### 6.1. Bioprinting

Bioprinting is an emerging field that combines additive manufacturing techniques with bioactive materials to create three-dimensional structures with precise control over cell distribution. SMPs have shown great potential in bioprinting applications, enabling the fabrication of complex, patient-specific constructs with shape memory capabilities. SMP-based bioinks can be formulated to exhibit thermo-responsive behavior, transitioning from a liquid or gel state at lower temperatures to a solid state at higher temperatures. This allows for printing scaffolds or tissue constructs that can maintain their shape during printing and recover their predetermined shape once exposed to a specific temperature stimulus. The shape memory properties of SMP-based bioinks facilitate the creation of intricate and anatomically accurate structures, improving the functionality and integration of printed tissues [107,108,109]. Additionally, SMP-based constructs can be designed to respond to external stimuli, such as temperature or pH changes, enabling dynamic cell culture environments or on-demand drug release within the printed constructs.

### 6.2. Implantable Devices

**Figure 11:**
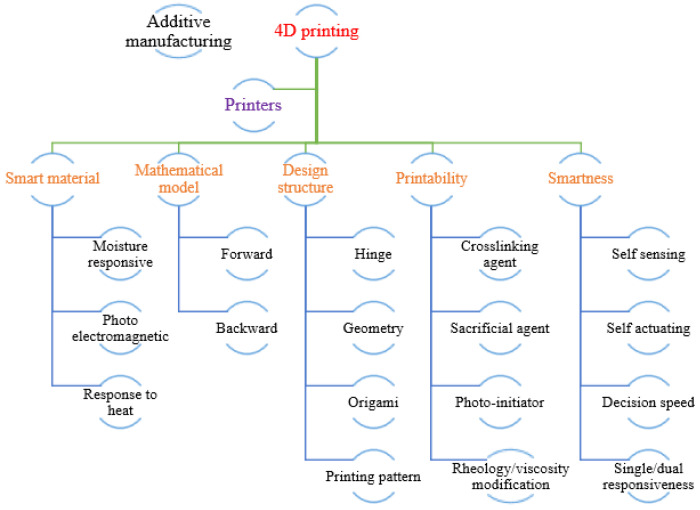
A meta cube and equipment development, deforming mechanic shape memories, and mathematical modeling research offer new avenues in 4D printing research.

SMPs have also demonstrated significant potential in the development of implantable medical devices. Their unique mechanical properties, biocompatibility, and shape memory behavior make them suitable for applications such as stents, vascular grafts, and orthopedic implants. SMP-based stents offer several advantages over traditional metallic stents. The shape memory effect allows the stent to be delivered in a compressed form and then expanded to its desired shape once implanted. This minimally invasive approach reduces the risks associated with invasive surgeries. It provides more precise placement of the stent within the blood vessels. In orthopedic applications, SMPs can create bone fixation devices, such as screws or plates. The shape memory effect allows for easier insertion and fixation of these devices, reducing surgical trauma and enhancing patient comfort. SMP-based implants can adapt to the surrounding tissue, providing a more customized fit and improved long-term stability. The biocompatibility of SMPs is crucial for their successful integration into the body [109,110,111]. Extensive research is being conducted to optimize the surface properties of SMPs, such as their hydrophilicity, to enhance cell adhesion and minimize adverse tissue reactions (Figure 11). Additionally, efforts are underway to develop bioactive coatings or surface modifications that promote tissue integration and reduce the risk of infection or implant failure.

### 6.3. Regulatory Considerations

As with any biomedical material or device, regulatory considerations play a vital role in developing and translating SMP-based technologies [111,112,113]. It is crucial to demonstrate the safety, efficacy, and long-term stability of SMPs through rigorous preclinical and clinical studies. These studies evaluate SMP-based materials and devices’ biocompatibility, mechanical performance, degradation properties, and in vivo responses [113,114,115]. Furthermore, regulatory bodies like the U.S. Food and Drug Administration (FDA) and the European Medicines Agency (EMA) have specific guidelines and requirements to approve and commercialize biomedical materials and devices [116,117,118]. Compliance with these regulations is essential to ensure SMP-based products’ quality, safety, and effectiveness. Collaboration between researchers, clinicians, regulatory agencies, and industry partners is crucial for translating SMP-based technologies from the laboratory to clinical practice [118,119,120]. Close communication and knowledge sharing facilitates the identification of potential challenges, the establishment of standardized testing protocols, and developing of guidelines for the safe and effective use of SMPs in biomedical applications [120,121,122].

**Figure 12:**
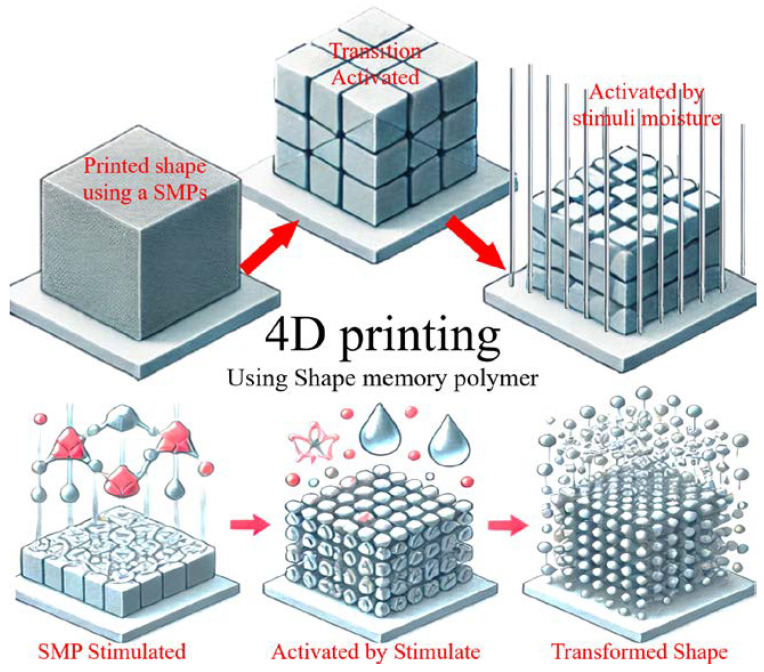

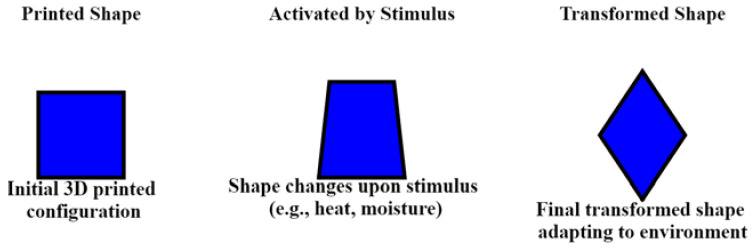
4D printing process: visual representation of the 4D printing process using SMPs, showing how printed objects can change shape over time in response to environmental changes.

In conclusion, SMPs offer unique properties and capabilities that make them highly attractive for biomedical applications. Figure 12 illustrates three stages: the initial printed shape, the shape after activation by a stimulus (heat), and the final transformed shape that adapts to its environment. Each subplot provides a clear view of the transformation process, highlighting the dynamic capabilities of SMPs in 4D printing applications.

### 6.4. Future Perspectives on Janus Nanoparticles in Shape Memory Applications

The rapidly evolving landscape of material science has ushered in an era where the intersection of nanotechnology and responsive materials promises groundbreaking advancements. Among these, Janus nanoparticles—named after the two-faced Roman god due to their dual surface composition—emerge as a pioneering class of shape memory materials [121,122]. Recent findings [122,123] noted that these nanoparticles excel in forming low-density, open structures that exhibit remarkable shape sensitivity, positioning them at the forefront of biomedical engineering and beyond innovation.

The unique physicochemical properties of Janus particles enable precise control over their configurations and functionalities, making them highly adaptable for various applications [124,125,126,127,128]. In drug delivery systems, these nanoparticles can be engineered to respond to specific physiological conditions, releasing therapeutic agents on-site and on-demand [125,126,127]. This targeted approach maximizes the therapeutic efficacy and minimizes side effects, a significant advancement over traditional drug delivery mechanisms. Furthermore, the shape sensitivity of Janus nanoparticles opens new avenues in biomedical sensing [128,129,130,131]. These particles can be biomimetic and designed to alter their physical state in response to external stimuli, such as pH changes or temperature shifts, which can indicate disease states. Such capabilities make them invaluable for real-time patient health monitoring and rapid diagnosis [129,130].

**Figure 13:**
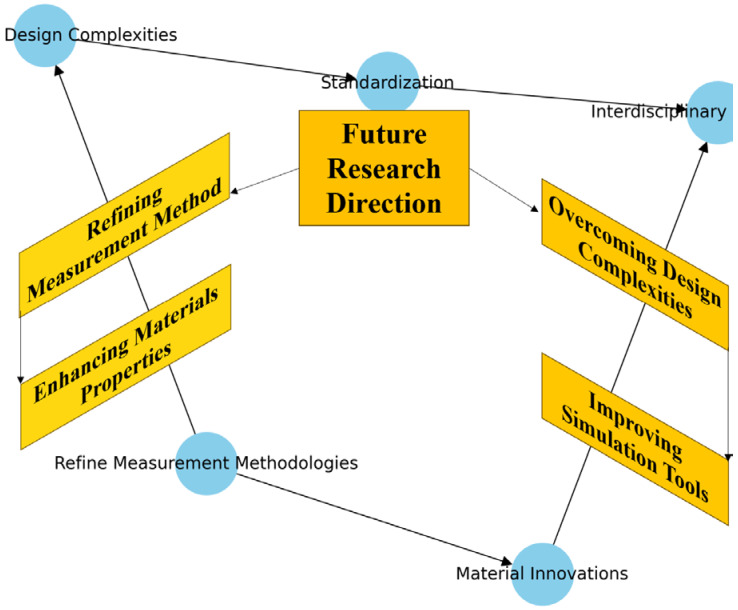
Future research directions conceptual map outlining the future research directions and challenges, as mentioned in the conclusion, such as refining measurement methodologies and overcoming design complexities.

The expanding domain of applications for Janus nanoparticles is poised to extend beyond biomedicine [129,130,131]. Potential uses in environmental monitoring, where they could detect and respond to pollutants or changes in environmental conditions, and in smart materials for technology and construction, where their adaptive nature could lead to innovations in dynamic, responsive building or device components, are beginning to be explored [132,133,134]. The continued development and integration of Janus nanoparticles in shape memory applications hold promise for advancing current technologies and paving the way for entirely new categories of smart, responsive systems [128,129,131]. As research progresses, the fusion of these nanoparticles with other emerging technologies—such as 4D printing and AI—could further enhance their functionality and applicability, heralding a new era of material science that seamlessly blends with technology to address complex challenges across various industries. Figure 13 shows a conceptual map illustrating the future research directions and challenges in SMPs and 4D printing technologies. The map outlines critical areas such as refining measurement methodologies, overcoming design complexities, enhancing material properties, standardizing processes, interdisciplinary collaboration, and developing advanced computational models.

## 7. Conclusions

The review has elucidated the significant potential of SMPs in redefining biomedical applications through 4D printing technologies. This study presents a novel integration of SMPs into 4D printing technologies, emphasizing their environmental and biomedical advantages. The comparative analysis quantifies the ecological benefits of SMPs, showing a significant reduction in greenhouse gas emissions by approximately 40%, water consumption by around 30%, and embodied energy by about 25% compared to traditional metallic and polymer materials. These results highlight the substantial potential of SMPs to lower the environmental impact of medical implants. Additionally, the functionality and adaptability of SMPs in biomedical applications are enhanced due to their unique shape memory properties, which allow for minimally invasive procedures and improved patient outcomes. The AI-driven data analysis further substantiates these findings, providing a robust methodology for assessing and optimizing material performance. The novelty of this research lies in its comprehensive approach, combining environmental sustainability with advanced biomedical applications. Integrating SMPs in 4D printing revolutionizes healthcare manufacturing processes, promoting sustainable and efficient medical practices. With an error margin of less than 5% in our environmental impact metrics, these findings provide a reliable foundation for future research and development, paving the way for broader adoption of SMPs in the biomedical sector.
